# Genito-Urinary and Syphilitic Diseases

**Published:** 1896-03

**Authors:** Byron H. Daggett

**Affiliations:** Buffalo, N. Y.


### GENITO-URINARY AND SYPHILITIC DISEASES.

Conducted by BYRON H. DAGGETT, M. D., Buffalo, N. Y.

### CYSTITIS IN CHILDREN.

ESCHERICH (*Jahrbuch Kinderheit Kunde*, 1895, *Archives of Pediatrics*, January, 1896,) reports ten cases of cystitis in girls under ten years of age. The symptoms were present from five to eight days before treatment was commenced and consisted of desire to urinate, slight burning upon passing urine and of pain in the region of the bladder. The urine was scanty, and upon standing deposited a copious sediment.

The microscope showed this to contain pus cells, bladder and kidney epithelium. Cultures made from the fresh urine showed unmistakably the presence of bacterium coli. In these cases urethral blennorrhea preceded the cystitis.

---

### CRYPTOGENIC CYSTITIS AND PYELITIS.

Posen (*Journal of the American Medical Association*, *August 3, 1895*,) states that cystitis often occurs in patients who have never been catheterised and pyelitis without being preceded by cystitis. Such cases indicate that pus-producers have immigrated from the circulation. Pyelitis frequently appears after abscesses and furunculoses. The healthy body continually harbors immense numbers of pus-producers, which relatively slight causes can send into the blood and kidneys.

Intestinal bacteria, prominent among them the bacterium coli, are innocuous parasites so long as they inhabit their normal domiciles ; but as soon as they migrate into other tissues they act as most energetic pus-producers. Experiments show that from eighteen to twenty-four hours after tying the rectum the entire body is inundated with the bacterium coli.

The bacillus prodigiosis was injected into the rectum of animals and the gut was then ligated ; after the lapse of the same time, this organism was found in all of the organs of the body and especially in kidneys and urine. Coloring matter in solution injected into the rectum under similar circumstances was found in the kidneys and urine within from twelve to fifteen minutes.

---

### THE IMPORT OF CASTS IN THE URINE.

Brewer (*Canadian Practitioner*, October, 1895,) says a person continuously or periodically passing urine containing casts, with or without albumin, is not in good health. His kidneys are vulnerable and he is prone to contract other diseases. The initiative process may not be an actual state of inflammation and there may not be recognised signs of kidney disease and yet the subjective symptoms may be very pronounced, unaccountable to the attending physician because sufficient attention is not attached to the presence of casts.

In some cases albumin is intermitting in its presence and appears only when a severe nerve storm rages. Both casts and albumin may be intermitting and there be present a dormant pathological condition, which may at any time be aggravated into activity. There are several reasons why the search for casts may be negative :

1. Microscopical incapacity.
2. Casts may be absent owing to latency of the kidney disturbance.
3. Insufficient instrumental equipment. The centrifuge should always be used.
4. The examiner may not attach sufficient importance to the presence of casts if albumin be not found.

Brewer alleges that casts found in the urine of athletes after great muscular exertion indicate damaged men. The symptoms produced by vulnerable kidneys are the various neuroses, particularly neurasthenia, gastro-intestinal and respiratory manifestations, migraine and other forms of headache.

---

### URINARY EXAMINATIONS.

Lichty (*Medical News*, November 9, 1895,) says : 1. A continued low specific gravity must be looked upon with grave suspicion, until it can be proved beyond doubt that the kidneys are normal.

2. In nephritis, especially of the chronic interstitial type, it may happen that at times during the greater part of the disease the urine may contain no albumin that can be detected.

3. Casts may be present in the urine when it is impossible to detect any albumin by the usual tests.

4. Casts are very easily destroyed in the urine by bacteria during the process of fermentation and unless the examination is made within an hour or two after the urine is passed, the failure to find casts does not prove the nonexistence of nephritis. The urine should be more frequently examined, especially after sickness.

---

### CALCULUS IN THE KIDNEY.

Bartholow says that borotartrate of potassium is the first remedy for calculus in the pelvis of the kidney. A weak solution must be used for a long time, a strong solution being detrimental. The calculus of the kidney is formed from uric acid and the neutral phosphatic alkaline salts are the best solvents of uric acid ; therefore, to promote its elimination they would appear to be the best remedies to administer. The fruit acids are very useful, therefore abundance of fresh fruit would also be indicated for the relief and prevention of nephritic calculi.

---

### A TEST FOR PUS IN THE URINE.

Drop a few minims of tincture guaiac into a test-tube partially filled with urine, heat it to about 100° F. and if it turns pale blue, pus is present in the urine. This is a convenient test, but not reliable, as the reaction sometimes fails to materialise, although pus is found by the microscope.

---

### A NEW CURE FOR SENILE HYPERTROPHIC PROSTATE.

Dr. A. Cully writes to the *New York Medical Record* that injecting cocaine twice a week for two months directly into the testicles, will cause the prostate to shrink and that this procedure is fully as effective as castration.

---

### ABORTIVE TREATMENT OF GONORRHEA.

Diday says: If the secretion is slight in quantity, slightly colored and more mucoid than purulent, if the lips of the meatus are not swollen and but slightly tinted with an erythematous redness, it is not too late to apply abortive treatment.

### A PRESCRIPTION FOR GONORRHEA.

J. William White gives the following prescription for injection in the second stage of gonorrhea :

| | | |
|---|---|---|
| ℞ | Mercuric chloride | $\frac{1}{6}$ gr. |
| | Carbolic acid | $1\frac{1}{2}$ drs. |
| | Zinc sulpho-carbolate | 24 grs. |
| | Boroglycerin (50 per cent. solution) | 2℥ |
| | Rose water sufficient to make | 8℥ |

### A SOLUTION FOR FEHLING'S TEST.

Dissolve 8.7916 grams of electrically deposited copper in 93 grams of concentrated sulphuric acid, diluted with an equal volume of water, and then add sufficient concentrated water of ammonia to bring to the measure of 1,000 c.c. ; of this solution, 10 c.c. represent 0.05 grain of glucose.

### NEPHRITIS VIEWED FROM THE STANDPOINT OF INDIVIDUAL CELL LIFE.

Meminger and Evans (*Medical News*, 1895, lxvii., p. 480,) say that physicians treating symptomatology and overlooking primarily diseased organs, fail to relieve their patients ; that is, they do not take into consideration the disturbance of the ultimate cell structure of which the organs are composed.

The cell itself is an organ and its function is as well defined as that of the major organs, which are but aggregation of cells. The cell performs its functions under normal conditions, unless perverted by irritation. Health depends upon normal cell action. Nephrites are generally considered diseases of the kidneys, followed by various constitutional changes.

Is nephritis or albuminuria primarily a disease of the kidneys ? Primarily, nephritis is acute or chronic irritation of the cell organs of the whole body, causing cell vomiting or the elimination of cell food, that is, albumin. Disease is irritation. Irritate any of the organs of the body and there arises peculiar manifestations. If this is true of large organs, is it not true of individual cells ? Irritants are brought in contact with cells causing perversion of function, inability to utilise nutritive material and it is thrown off or vomited. The stomach rejects crude food, the cells reject peptonoids and albuminoids. Numerous diseases and various drugs cause specific irritation of the cell organs of the entire body.

The tendency of acute inflammation is to chronicity, and treatment should be directed to the affected part. The individual-cell organs will as surely become chronically inflamed as the major organs. Acute gastritis causes acute vomiting; acute cell-irritation causes acute cell-vomiting or acute albuminuria. From this standpoint albuminuria is a direct symptom of either acute or chronic general cell-irritation; the hypersensitive cells throw off their food material, albuminuria and kidney changes are secondary thereto. Now, assimilated food material and all systemic waste material must be eliminated and the kidneys are the principal source for sifting these products from the blood, and if overworked they suffer.

Treatment should be directed to remove the cause and soothe the general cell-irritation, promote hygienic conditions, mental and physical rest, together with proper diet. Milk is nonirritating, therefore the best food. Rare meats without condiments, except salt, and all the cereals and white potatoes may be allowed. Nitrogenous foods may be allowed if the albuminoids are assimilated. Opium in small doses allays irritation and may be given for a long time. Codliver oil improves the nervous system, increases urine and perspiration, improves the appetite and nutrition, increases the number of the red blood-cells and healthy cell formation and has a general alterative effect.

